# iSupport for Young Carers: An Adaptation of an e-Health Intervention for Young Dementia Carers

**DOI:** 10.3390/ijerph20010127

**Published:** 2022-12-22

**Authors:** Patricia Masterson-Algar, Kieren Egan, Greg Flynn, Gwenllian Hughes, Aimee Spector, Joshua Stott, Gill Windle

**Affiliations:** 1 School of Medical and Health Sciences, Bangor University, Bangor LL57 2EF, UK; 2 Department of Computer and Information Science, University of Strathclyde, Glasgow G11 XH, UK; 3 Department of Clinical, Educational and Health Psychology, University College London, London WC1E 6BT, UK

**Keywords:** young carer, dementia, e-health, co-design

## Abstract

Young dementia carers need to be recognised and supported in their role. They need help to understand the illness, what changes are expected and how it can affect their family member. Many support services, partly due to the COVID pandemic, have moved online and have been shown to be acceptable as they are low cost and reduce access barriers. iSupport is an evidence-informed e-health training programme developed by the World Health Organization (WHO) to support adult dementia carers. This paper reports on the co-design of an adapted version of iSupport for young carers. A theoretically driven co-design approach, drawing on the lived experiences of young dementia carers and experts who work with this target group was followed. As a result of this study iSupport for Young Carers was created. It is the first e-health intervention of its kind and aims to support the mental health, knowledge and skills of young dementia carers. In turn, it could improve the quality of the support that service providers can offer, and this can result in increased levels of identification of these young people. The work presented also provides opportunities for other countries and demographic groups to translate and adapt iSupport for Young Carers to their specific cultural context.

## 1. Introduction

Approximately 850,000 people in the UK are living with dementia of whom, 44,000 have been diagnosed with young onset dementia [1,2]. This overall number is predicted to increase to over 2 million by 2051 [3]. In much of the world, including the UK, the majority of people with dementia receive care at home from a family member [4,5,6]. The number of children and adolescents who find themselves carrying out caring responsibilities for a family member with dementia is also rising [2,7,8,9,10]. There are two main reasons for this. Firstly, more and more families are taking care of a grandparent [10,11] with dementia. Secondly, younger people with dementia are more likely to be a parent to dependent children, particularly as people are waiting longer to have children.

These young people under the age of 18 who undertake unpaid care are defined as ‘young carers’ [12,13]. Providing care for a family member is associated with both positive and negative outcomes for young carers [14,15]. Positive outcomes include improved self-esteem, resilience, and maturity [10,16]. However, the caring role is also associated with negative wellbeing outcomes such as anxiety, stress and isolation which will impact on all aspects of a young person’s life such as schooling or personal relationships [14,16,17,18]. Despite this evidence and even though over the past decade, young carers in the UK have acquired specific legal rights and provisions reflected in statutory guidance and national policy [19,20] this group remains under supported and under researched. Young carers rarely receive appropriate training or support for their caring role [11,13,21]. The scarcity of specialist services and the dearth of research literature is more accentuated when talking about young dementia carers [2,9,10], whose needs, due in part to low level of self-recognition in this group, are often overlooked leading to feelings of abandonment [2,16,21]. Evidence is accumulating, advocating the need for formal support systems to recognise these young people and support them so they can understand the illness, how it affects their family member and what changes are expected [2,10,11].

Educational programs that focus on knowledge of dementia, confidence in caring tasks and coping strategies to handle emotions have been shown to improve the wellbeing of carers and care recipients [22]. In recent years, and accelerated by the COVID pandemic, many support services have moved online [23,24]. Evidence shows that these interventions that are often free to access and self-monitoring are more accessible and acceptable, particularly to the younger generations [25]. They reduce geographical boundaries to access support and are low cost, hence they are often linked to higher uptake [24,26,27,28].

iSupport is an evidence-informed e-health training programme developed by the World Health Organization (WHO) to help adult dementia carers take care of themselves whilst providing quality care [29,30]. The initial version of iSupport was developed in English and has been through transcultural adaptations in several countries, such as Portugal [31], Australia [32] and India [33]. However, all adaptations to date have targeted adult dementia carers. Modifying an intervention, considering the context and the needs of the target population, is key to the success of scalable implementation and uptake as it can improve their attitude towards it [28,34,35]. Drawing on the lived experiences of young dementia carers and experts who work with this target group, this paper reports on the co-design of an adapted version of iSupport for young carers. We report on this adaptation process, the outcomes and the lessons learned.

## 2. Materials and Methods

**Figure 1:**
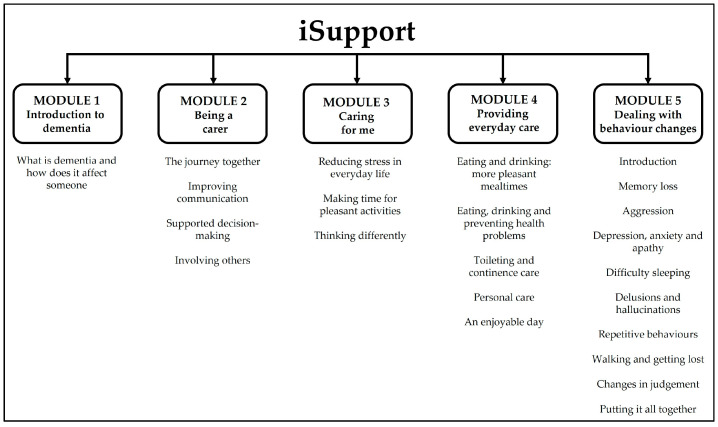
iSupport content.

iSupport is an e-health intervention informed by evidence from psychoeducation, cognitive reframing and person-centred care theories, it consists of 23 lessons distributed across five modules (see Figure 1): Introduction to dementia; Being a carer; Caring for me; Providing everyday care and Dealing with behaviour changes [29]. The lessons start with a short summary describing its purpose. They then comprise relevant information, illustrative images, caregiving scenarios and interactive skills training exercises. Users can access the programme anytime/anywhere, as often as they wish and free of charge.

### 2.1. Methodological Approach

The adaptation of iSupport followed a co-design process described in the WHO adaptation guide which is available to all licensees [36] and has been successfully applied in other adaptations of iSupport [37,38]. This guide outlines a number of stages to be followed which involve the active participation and input of the ‘experts by experience’, via a series of workshops. By following a co-design approach [16], we ensured the creation of an adapted version that is equivalent to the generic in its theoretical underpinnings and internal logic while being culturally relevant to the target group, in this case young dementia carers. In order to ensure in-depth exploration of all core factors we were also informed by the Ecological Validity Model [EVM] [39]. This model is a methodological tool that has been widely used to carry out cultural adaptations of psychosocial interventions [28]. The EVM suggests the exploration of a number of dimensions such as language, content, and context in order for the intervention to be acceptable to the new target group.

### 2.2. Participants

Recruitment took place between September – October 2021. Purposive sampling was used to identify 1) 4–6 professionals with experience of working with young people and 2) young dementia carers (n = 6–8). Young carers between the ages of 11 and 17 were recruited through stakeholders’ networks, social media and national carers and young people’s associations (Exclusion and inclusion criteria are presented in Table 1). All participants (and their parents if younger than 16) who expressed an interest in taking part in the study were sent a participant information sheet (PIS) and a consent form. They were also invited to meet the researcher (PMA) via an online Zoom meeting (or telephone conversation) to assess eligibility and ask any questions about their (or their child’s) involvement in the study. During this conversation PMA also discussed sensitive content of iSupport and how it could affect young participants.

### 2.3. Data Collection

Basic demographic information was collected during the initial Zoom conversation (or telephone conversation) from all young people recruited into the study (e.g., age, gender, school year, postcode, and brief family context). PMA shared a document (via email and by post) with instructions on how to access the generic version of iSupport (hosted by the Pan American Health Organization platform) with all participants. At that same time they were also provided with a printed pdf version of the iSupport manual.

**Figure 2:**
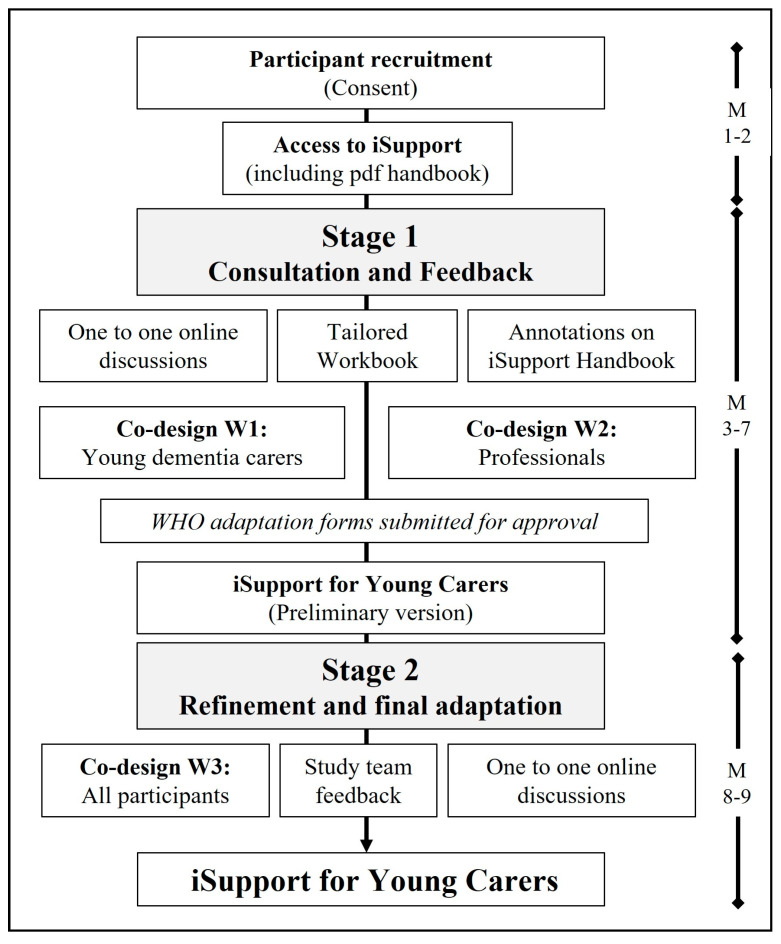
Adaptation process including timeline (M: month).

Following the WHO adaptation guidelines, the work was conducted in two stages (see Figure 2): (1) Stage 1: Consultation and feedback – preliminary adaptation; (2) Stage 2: Refinement and final adaptation.

#### 2.3.1. Stage 1: Consultation and Feedback–Preliminary Adaptation

The aim of this first stage was to collate high quality experiential information and feedback from participants. In order to ensure this was achieved, data were collated via the following methods, which participants could choose from depending on their preferences:

▪ The completion of a ‘tailored made’ workbook (only young carer participants) – this included all sections of iSupport, and it was created following the template of the WHO adaptation guideline documents [36]. Young carers completed the workbook in their own time as an when convenient. The workbook allowed participants to, for each section, select whether they would ‘*Take this section out*’, ‘*Keep it in as it is*’ or ‘*Keep it but making changes to the language, content, or illustrations*’. Free text space was also provided for participants to write down ideas on how they would improve that section. Participants could choose whether they completed the workbook on the computer (Word document) or using a printed hardcopy.
▪ Online face to face feedback sessions with PMA. These meetings with young carers and professionals were audio and video recorded with the consent of participants and handled in accordance with the UK Data Protection Act [40]. Young carers followed the sections of the workbook and discussed their annotations with PMA. Meetings with professionals were not structured and focussed on particular sections of iSupport identified by them as more in need of adaptation.
▪ Annotations on the iSupport pdf handbook (young carer and professionals). Participants were told that these annotations could be regarding all aspects to iSupport including content, structure, design, or language (the iSupport handbook is free to download at https://www.who.int/publications/i/item/9789241515863 (accessed on 21 December 2022))

All participants were given a minimum of two months to look at the materials and collate their feedback. After this period participants were asked to return their completed workbooks by pre-paid post, or to photograph and email any annotations on the handbook to PMA.

PMA collated and analysed all feedback. We used this information to organize two co-design workshops. These workshops followed the principles of experience-based co-design in which all participants are involved as knowing subjects who bring their perspectives into the knowledge-production process [16]. All co-design workshops were led by PMA, held remotely and recorded in Zoom.

*Workshop 1 with young carers:* Prior to the workshop all participants received a ‘toolkit’ by post which included materials that would be used on the day (e.g., Play Dough, white board, and colour pens). PMA led the workshop and member of the research team and co-author (GH) who has experience being a young dementia carer was also present. During the first part of the workshop, participants had the opportunity to relate to the group their general thoughts regarding iSupport. The second part utilised mind maps and charts to explore young carers’ support networks and lived experiences of caring (please see Supplementary File S1 for more details on the content and structure of Workshop 1). The main purpose of the workshop was to apply a co-design approach to investigate experiences of caring to generate rich and meaningful examples that could be added to the iSupport platform. A professional illustrator, hired by the study, attended the workshop as an ‘observer’. She also presented initial drafts of new illustrations for young people to comment on.

*Workshop 2 with professionals:* The structure of this workshop was similar to that of Workshop 1 and professionals had a chance to report on their overall thoughts regarding iSupport. However, prior to this workshop, participants were provided with a summary document that was informed by young carers feedback and Workshop 1, outlining particular sections of iSupport where there were discrepancies amongst young carers on possible adaptations. During the second part of the workshop these sections were discussed in detail to reach agreements on the way forward. Professionals were also able to provide feedback on the proposed new illustrations.

All Stage 1 feedback was collated by PMA and a complete set of adaptations was agreed by the research team and submitted to the WHO for approval. Once approval was granted, PMA worked with Bangor University’s IT department and a Digital Learning Designer to produce a preliminary version of iSupport for Young Carers (iSupport for YC), implementing the adaptations in the e-learning course creator tool Articulate Storyline 360^©^.

#### 2.3.2. Stage 2: Refinement and Final Adaptation

The preliminary version of iSupport for YC was shared with all participants. Using the editing tools in Articulate Storyline 360^©^ a link was generated for each of the iSupport for YC modules. All participants were given three weeks to access the links and post comments/provide feedback online. After this period all feedback was collated, and Workshop 3 was organised.

*Workshop 3 with young carers and professionals:* The primary aim of this final co-design workshop was to allow everyone to share their opinions and suggestions on the preliminary version of iSupport for YC, in order to finalise the proposed adaptations. A secondary aim of this workshop was to gather participants opinions on ways in which they thought iSupport for YC could possibly have an impact (or not) on them as young people with caring responsibilities. These discussions would help inform future choices of outcome measures at the time of evaluating the feasibility of iSupport for YC. Participants who were not able to attend Workshop 3 were offered a one-to-one online meeting or the possibility of providing feedback over email.

### 2.4. Data Charting and Analysis

All feedback data collected during Stage 1 was collated and reviewed to generate a set of proposed adaptations. PMA revisited and selectively transcribed recordings from workshops and one-to-one meetings to gain familiarity with the data. All data were charted using iSupport WHO adaptation spreadsheets. These included all sections of iSupport and allowed all proposed adaptations and the rationale supporting them to be charted in a standard format. Additionally, following a deductive approach, proposed adaptations were grouped according to the dimensions suggested in the EVM. Similarly, all data collected in Stage 2 (workshops and one to one meetings) were selectively transcribed and reviewed by PMA to inform further adaptations of the preliminary version. At this stage the preliminary version was shared with members of the study team with clinical psychology expertise and final feedback was collated and acted upon. As a result, the final version of iSupport for YC was produced.

Ethical approval was granted by Bangor University’s School of Medical and Health Sciences Academic Ethics Committee (REF 2021-16915). Before initiating data collection, all participants read the PIS, were given a chance to ask questions and provided informed consent.

## 3. Results

### 3.1. Participants

A total of nine young carers showed an interest in the study. Three of them did not meet the eligibility criteria as they were older than 17. The remaining six young people of ages between 12 and 16, met all criteria and were recruited to take part in the study (Table 2). Amongst these, five were girls and one was a boy. Three of them helped care for their grandparent and the rest helped care for their parent living with dementia. At the time of the study all participants lived with their family member with dementia. All participants were in secondary school in full time education. Out of the six participants, five had received limited support for their role as young carers. None of them had a heavy load of caregiving hours.

Nine professionals showed an interest in the study, however five of them consented to take part in the study. One withdrew during Stage 1 due to personal commitments. Among the four remaining participants, two were charity workers with experience of working with young carers. Two were experienced dementia researchers whose topics of research involved younger populations.

### 3.2. Stage 1 and Stage 2 Findings

**Figure 3:**
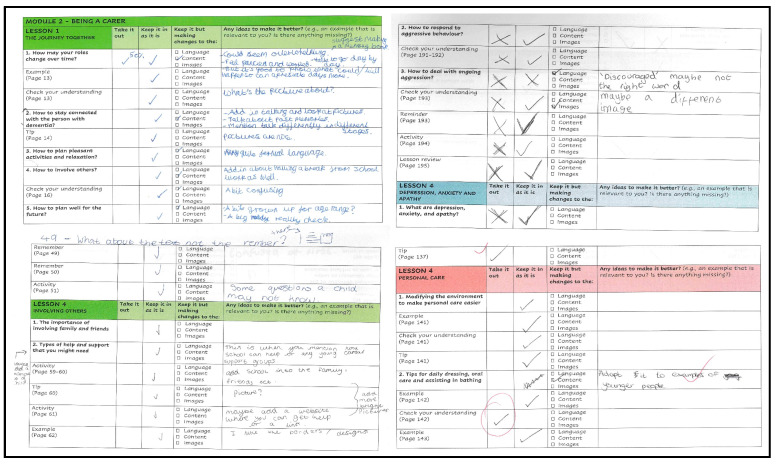
Example of young carers feedback (workbooks).

Five young carer participants returned their completed workbook by post (see Figure 3 for examples). In addition, two of these young carers chose to provide further feedback via weekly Zoom face to face meetings with PMA and GH. A total of eight one to one meetings, lasting between 20–30 min each were carried out. One young carer only provided feedback during Workshops 1 and 3. None of the young carer or professional participants provided feedback via annotations on the iSupport pdf handbook.

All six young carers attended Workshop 1 which lasted 2 h. Two professionals attended Workshop 2. Due to work commitments and personal circumstances the remaining professional participants preferred to provide feedback via email or during one-to-one online meetings with PMA. Finally, four young carers and three professionals attended Workshop 3. Overall, participants who took part in workshops were keen to contribute and happy to share their insights and experiences.

#### 3.2.1. Adaptation

Following the WHO adaptation guidelines and informed by the EVM, a number of adaptations were carried out based on the feedback from the young carers and professionals. These adaptations were grouped in the following four dimensions: structure and design; context; content (illustrations, narratives—people); and language (Table 3 presents examples of issues identified in the feedback and adaptations to address these). By focussing on these dimensions, we intended to increase the relevance, the acceptability and comprehensibility of iSupport while keeping the completeness of the theoretical premises underpinning it.

##### Dimension 1: Structure and Design

Overall, participants’ feedback regarding the structure of modules and lessons in iSupport (as detailed in the introduction) was positive. Both groups, young carers and professionals considered this structure logical and easy to follow. Hence, no adaptations were needed.

However, several issues related to the design and ‘look’ (delivery) were identified as in need of adaptation. All participants considered that the design and presentation of iSupport was not appropriate or appealing to young people as it was described as ‘boring looking’ and ‘overcrowded’. Hence, several adaptations proposed by study participants were carried out. Firstly, the quantity of text was reduced as much as possible without compromising its readability and meaning. Long paragraphs were shortened and whenever possible their content presented as ‘bullet points’. Secondly, to increase clarity and appeal, the font of titles was increased in size and made ‘bold’. Additionally, coloured text boxes were used to provide contrast and separate concepts. Thirdly, a different colour scheme was given to each Module to help differentiate them. Brighter tones were chosen. Fourthly, new illustrations were added throughout to ‘breakdown’ the text. Finally, to improve the delivery of the multiple relaxation exercises included as part of Module 3, a ‘read aloud’ option was added which meant participants could listen to the exercises and follow the steps rather than reading them.

##### Dimension 2: Context

A number of contextual characteristics were identified by participants which were particular to this adaptation for young carers. The needs and lived experience of the young population we were targeting are unique and influenced by their developmental stage and transition to adulthood. iSupport for YC had to reflect participants’ and professionals’ views regarding, firstly, the need to support young people, protect and safeguard them from inappropriate and highly demanding caring responsibilities, and secondly, the need for their role as young carers to be normalised and accepted rather than being linked to shame and stigma. Hence, several adaptations were agreed (Table 3). ‘Remember’ and ‘Keep in Mind’ coloured text boxes were added throughout the programme reminding young people that they ‘*shouldn’t have to deal with caring on their own*’ and that they ‘*should reach out for help if things were getting too difficult or they felt they are not coping*’. Additionally, reflecting participants’ feedback, several sections were deleted (e.g., information on sexual relationships) and others reduced in content as, whilst deemed important, young people identified them as tasks that they could help with but should not be in charge of (e.g., continence care). Throughout iSupport for YC edits were made to include information on types of support that young carers could reach out to. For example, schools, friends and family members or charities that support families affected by dementia and young carers in particular.

Finally, some sections of iSupport for YC were edited and new sections were added to reflect a context which normalises young carers and their lived experiences. This was achieved by using expressions such as, for example, ‘*it is normal to feel this way’* or ‘*many people feel this way*’ (Table 3).

##### Dimension 3: Content

**Figure 4:**
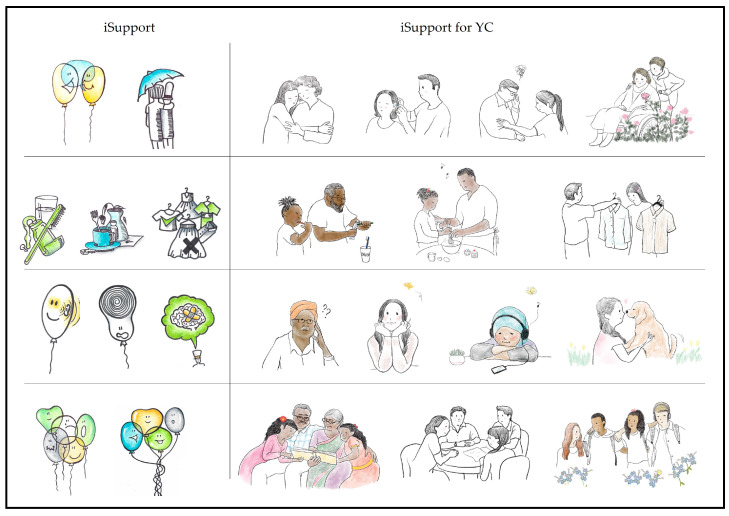
Examples of human characters replacing the original illustrations.

Illustrations: The illustrations in the generic version of iSupport were described by participants as irrelevant and in some cases patronising. In their feedback, young carers reported not understanding what illustrations meant or what their purpose was. All participants considered that it was important for the adapted version to include illustrations, as they played an important role in reinforcing messages and enhancing the acceptability and relatability of a tool. Participants expressed a preference for illustrations portraying human characters. As a result, the professional illustrator hired for the study created illustrations of young people with or without their family members with dementia (see Figure 4). The characters in these illustrations often emphasised the actions described within the carer case scenarios. Human figures represented a range of ages and ethnicities.

**Figure 5:**
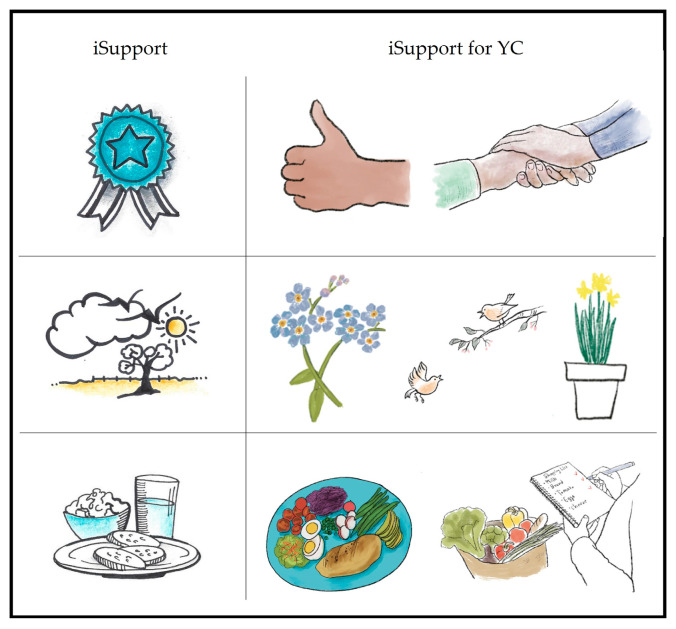
Examples of adaptations to illustrations.

In addition to human characters, meaningful symbols and other illustrations related to the content were added, some to replace current ones and others as new additions (see Figure 5). For example, ‘*forget-me-not’* flowers were scattered throughout the programme. Others such as a ‘holding hand’ or a ‘thumbs up’ were added to reinforce a message of ‘support’ and ‘achievement’.

**Figure 6:**
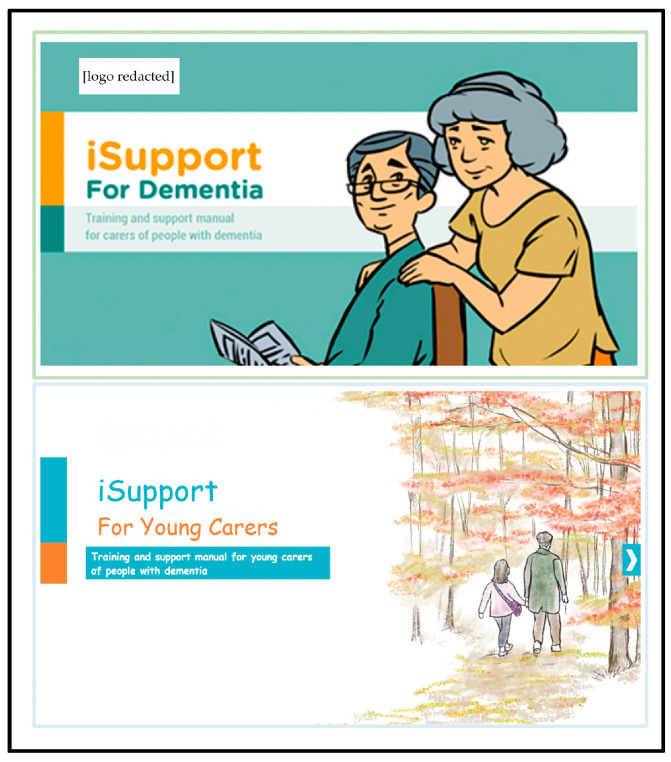
iSupport cover before and after the adaptation.

One important point that was discussed in detail with participants was the cover page, which all participants agreed was not appropriate for a young audience. As a result of participants’ feedback and after a number of iterations a new cover page was designed (see Figure 6).

Narratives (case scenarios): Every lesson in iSupport included ‘examples’ that described possible case scenarios that carers may experience. Participants considered that narratives and characters in the original iSupport were not acceptable or relevant to the young audience, as they were set in an adult context with the carers being adults, often spouses. Consequently, and informed by data from the Stage 1 workshops, case scenarios across the five modules of iSupport were edited and other new ones created to place the focus on a young character with caring responsibilities within a family unit. These characters were young people with a parent or grandparent living with dementia.

However, as both young carers and professional participants reported, it was important to address the fact that, ideally, young carers should not be expected to take on heavy loads of caring responsibilities, for example when dealing with personal care or behaviour changes. Hence, it was agreed that when these topics were presented in the form of a case scenario, the teenager character would be described as providing a ‘supporting’ role to the primary carer (see Table 3 for examples). It was considered that this adaptation would significantly increase the relatability and acceptability of the programme.

**Figure 7:**
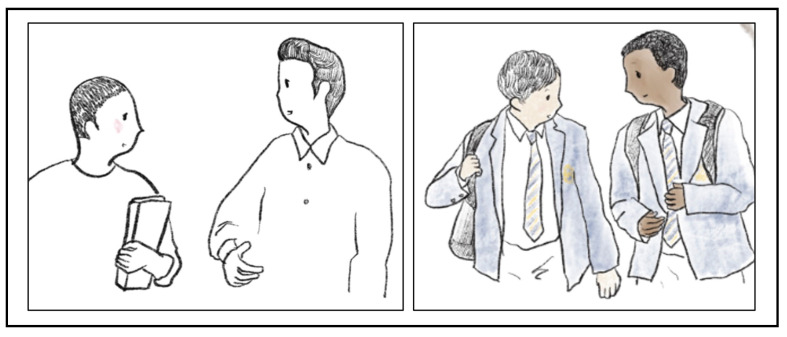
Examples of school-related illustrations.

Two further adaptations of the narrative were carried out as a result of participants’ feedback. Firstly, case scenarios and activities were edited to include references to teachers, support services within school and also to schoolwork as part of the daily routine of young people (see Table 3 for examples), and it was agreed that a new section ‘*How school can help*’ would be added to Module 2. In addition, to reinforce ‘school life’, illustrations portraying teenagers in school uniform or engaging in school related activities were added (see Figure 7).

Secondly, young carers discussed the lack of information in the original iSupport regarding the impact that changes in care can have on young people and their families. Hence, one of the young carers agreed to help write a new section called ‘*Planning for future care*’ (Module 2). This section discusses how people with dementia will often have to move into a care home and how this can be a sad and difficult time for families, using a case scenario. The section also provides strategies to deal with this change and the feelings it can generate (e.g., guilt, sadness).

##### Dimension 4: Language

Participants identified several issues related to the comprehensibility and acceptability of the language used in the original iSupport. In terms of comprehensibility, participants pointed out that, across all modules, some of the expressions and terms used were often complex and, at times, threatening or ‘scary’. This was particularly true in Module 1 (Introduction to dementia) and Module 5 (Dealing with behaviour changes). Hence, identified terms were replaced for lay, easier to understand terms (Table 3).

Feedback from professionals and experts identified the importance of using tentative language in the iSupport multiple choice case scenario exercises (Check your Understanding) in order to avoid the options sounding judgemental. As a result, the expressions ‘correct’ and ‘incorrect’ used in the original iSupport were replaced with expressions that would present answers as having the potential of being more or less helpful (‘the most helpful/unhelpful answer’). This adaptation avoided, firstly, the risk of young people losing faith in iSupport if for example the answer classed as ‘correct’ did not work for them and their family. Secondly, it prevented the young person feeling blamed if they choose to take a different approach. Finally, as a result of collated feedback, several further adaptations to the language were carried out which are presented in Table 3.

## 4. Discussion

This adaptation of the WHO’s generic version of iSupport is, to our knowledge, the first e-health tool of its kind addressing the needs of young people with caring responsibilities for somebody with dementia; a group to date under-researched and under-supported [2,10]. It provides a new resource to help support the mental health, knowledge, and skills of young carers. Globally, it provides opportunities for other countries to translate and adapt iSupport for YC for their specific cultural contexts.

The lessons learned during this adaptation process resonate with what previous studies, mainly focussed on older adult carers, have identified. Our findings advocate for young carers being viewed as active and contributing members of a family rather than observers and passive members [16,41,42]. As we know, caring for a family member is associated with both positive and negative impacts on young people’s lives [13]. However, we also know that for example, some of the positive outcomes, such as increased resilience and maturity [43,44] or empathy [45] are highly correlated with family cohesion and sufficient practical and psychological support [10,16,46]. iSupport for YC places young carers at the centre whilst reinforcing their role as contributing members of a family or network who need to be supported. Our adaptation work has resulted in a tool that moves away from viewing young carers as passive victims and reflects the vital role that all family members have in supporting each other.

As previously stated, evidence shows that dementia carers and young dementia carers in particular are under supported [10]. Additionally, that the COVID-19 pandemic has had a dramatic effect on their wellbeing [47] and has accelerated the development and move towards reconsidering the way in which support is designed and delivered [23]. Our results resonate with evidence reporting the fact that online support tools, such as iSupport for YC, are well received and accepted by young carers [25]. Our study participants appreciated the fact that this type of tool can be accessed from anywhere at any convenient time. Professionals taking part also reported that by not relying on face-to-face contact, it has the potential to help break down the barrier to these young people accessing support which is linked to the associated fear of being stigmatised and misunderstood [9,11,17]. We hope that, by including adaptations that help normalise the role of young dementia carers, iSupport for YC has the potential to increase the visibility of this group by helping young people feel comfortable identifying as a ‘young carer’ and accessing relevant services as a result.

By following an authentic co-design approach, we have been able to create a tool that is personalised to young dementia carers and their lived experience. This is vital to its success, as findings from recent reviews on e-health interventions for informal carers of people with dementia have reported [24,48,49]. We are confident that our personalised adaptation will support young carers in their role and will also contribute to the protection and safeguarding of this vulnerable group. This is vital as, in line with what professionals in our study reported, evidence shows that inappropriate responsibilities and/or caring load undermines their mental health and wellbeing and can exacerbate the negative impacts of caring [10,13].

The careful articulation of the adaptation methodology followed in this study provides a useful point of reference for researchers undertaking adaptations of interventions and could be applied to a wide range of target groups beyond young carers. We followed the WHO adaptation method and were guided by principles of co-design [16] and the EVM [39]. This ensured that the process of adaptation was rigorous and captured all relevant elements, which evidence has shown to be essential [28]. We received meaningful and valuable feedback from both young carers and professionals and held back on key design decisions until this was collated and analysed. Through this feedback, we were able to ensure that potential barriers to use around content, context and language were explored in depth and heavily embedded in the final product. On the one hand, the invaluable feedback from young carers heavily informed preferences for design, illustrations, and content regarding lived experiences. On the other hand, experts were best positioned to provide input related to behaviour and clinical aspects. This bottom-up co-design approach we are confident will maximise the validity of iSupport for YC.

### 4.1. Future Research

Adaptations like iSupport for YC need to be examined and evaluated [28,35,50]. Following from this work and in line with the UK Medical Research Council Guidelines for the development of complex interventions [51], we will complete a small-scale feasibility study to determine whether the intervention is feasible and acceptable to the target population. It will also help identify factors to be considered, and further adaptations needed in iSupport for YC to increase its sustainability prior to its scaling up and wider implementation [35].

Further research should follow, with the aim to investigate the effectiveness of iSupport for YC at supporting specific groups of carers such as those from minority ethnic backgrounds or of different ages, with different caring loads and with different relationships with the person with dementia they help care for. Equally important would be to explore the suitability of iSupport for YC at different stages of the disease, as evidence shows that it is not uncommon for adult carers to seek help only as the disease reaches its last stages [52]. Finally, we also advocate for further research focussing on investigating the acceptability of mixed web-based delivery methods, as recent evidence suggests that, for example, personalised contact (via web platforms) might increase adherence and hence be associated with greater health and wellbeing benefits of e-health interventions [24,53,54].

### 4.2. Limitations

We acknowledge that this study has several limitations. Firstly, our sample of young carers and professionals was small, although sufficient as per the WHO adaptation guideline recommendations. All young participants were introduced to the study by their parents who initially contacted PMA for more information. Hence, we can infer from that all young people who took part were supported by their families and were prepared to admit they were young carers. Four out of the six young carers had received support from young carers charities, although the extent of this support was very limited (reflecting the lack of support available to this group) and none of the young people had a heavy load of caregiving hours. As previously discussed, the load of caring, the severity of dementia of the family member as well as the amount of support that young carers receive will influence the way in which they experience the impact of caring (Santini et al. 2020). Secondly, although young carers were given a generous amount of time to provide feedback during the different stages of the study, the nature of this type of research is time-consuming. This could have impacted on the quality of the feedback, as participants were juggling their involvement in the study with other responsibilities as carers and as young students. Thirdly, we acknowledge that the quality of the feedback relied on participants being competent readers with a reasonable level of academic ability. Finally, the age of our participants ranged from 12–16 and we are aware that needs and experiences of 11-year-olds can be very different from those transitioning into adulthood at 16. Similarly, all but one participants were girls. We acknowledge the fact that gender might have a significant role in shaping experiences of caring and its impacts as it is the case in the adult carer population. All co-design workshops were delivered online which made it easier for participants, particularly young carers, to attend. Although PMA planned the content and discussions carefully to ensure everyone had a chance to contribute, we are aware that this type of delivery method might have had an impact on the quality of the findings. Whilst we acknowledge these limitations, we are confident that our participants were able to provide detailed experiential information that maximised and increased the relevance of iSupport for YC and that, thanks to the use of enough data collection strategies, we ensured that all participants were able to relate their experiences and voice their opinions in the way best suited to their wishes and abilities.

This study was conducted in the UK, a high-income country with a significant level of awareness on issues around young carers and a range of third sector organisations running ‘young carers projects’. We acknowledge this as a contextual limitation. However, in low-income countries, support is even harder to access and often non-existent [55]. Hence, we believe carers in these countries might benefit the most from a web-based tool such as iSupport for YC.

## 5. Conclusions

Countries such as the UK have made commitments, via policies and guidelines, to support the health and wellbeing of young carers by, for example, offering training to help them manage the challenges associated with their caring role. However, to date this support is very limited and often not fit for purpose. We are confident that iSupport for YC will provide a unique opportunity to address current gaps. It will also help service providers improve the quality of the support they offer and as a result awareness and identification of these young people has the potential to improve.

## Figures and Tables

**Table 1:** Participant eligibility criteria.

Inclusion criteria	**Young carers** 1) Young people between the ages of 11–17 who self-identify as a carer of a person with a confirmed diagnosis of dementia (through self-report of the carer). 2) The person they are caring for is not living in a full-time care facility. 3) They have been caring at least weekly for at least 6 months.	**Professionals** 1) Have regular contact with young people and young carers (e.g., teaching staff involved in pastoral care, young carer charity workers, social workers in children’s services)
Exclusion criteria	1) Receiving treatment from Child and Adolescent Mental Health Services at the time of recruitment. 2) Unable to comprehend written English. 3) No access to the internet. 4) Have previously used ‘iSupport’ materials in the last 12 months.	1) No regular contact with young people and young carers as part of their work. 2) Unable to comprehend written English 3) No access to the internet

**Table 2:** Characteristics of young carers (YC) who took part in the study.

	Age	Gender	Family Member with Dementia	Brief Family Context (at the Time of Study)
P1	12	Female	Father	P1 and P2 are siblings. They help care for their dad who was diagnosed with early onset dementia when they were very young. They have received limited support in their role as YC (e.g., peer support online groups).
P2	13	Female	Father	
P3	16	Female	Grandma	P3 and P4 are siblings. They support their parents in caring for their grandma who lives with them and is in the last stages of dementia. The family have support from a dementia charity and paid carers. P3 and P4 have attended online YC support groups.
P4	12	Female	Grandma	
P5	13	Female	Father	P5 helps her mum care for her dad who was diagnosed with early onset dementia when she was 3 yrs. old. She has an older sibling that has recently moved away to go to university. The family have support from a dementia charity and paid carers.
P6	14	Male	Grandad	P6 and her older brother help their mum care for their grandad who lives with them and is in the last stages of vascular dementia. The family have support from paid carers who visit four times a day. P6 has not received any support as a YC.

**Table 3:** Examples of feedback and adaptations according to the WHO adaptation guidelines and EVM framework.

Dimension	Feedback (Identified Issue)	Adaptation
**STRUCTURE** **& DESIGN**	Information presented in long paragraphs.	▪ Reduction in text (meaning remaining) ▪ Use of bullet points to summarize ideas and concepts ▪ Illustrations added to ‘break down’ the text
	Difficult to distinguish between sections	▪ Titles: Font size increased and made ‘bold’ ▪ Different background colour scheme (brighter choices) for each Module
	Difficult to identify important information within a section	▪ Addition of coloured text boxes
	Difficult to follow the information included in Module 3 (Lesson 2) which describes several relaxation exercises	▪ Read aloud option added to the relaxation exercises
**CONTEXT**	The way that the role of young carers is presented needs to be ‘normalised’	▪ Addition of expressions such as ‘*This is normal*’, ‘*Many people feel this way…*’, ‘*It is normal to…*’, ‘*For many people your age…*’
	Young carers need to be ‘protected’ from heavy caring loads and inappropriate caring responsibilities and this needs to be reflected in the text	▪ ‘Remember’ and ‘Keep in Mind’ coloured text boxes added throughout the programme reminding young people that ‘*you shouldn’t have to deal with caring on your own*’ and that they ‘*you should reach out for help if things are getting too difficult or you feel you are not coping*’ ▪ Young carer characters included in the case scenarios described as ‘helping care for a family member with dementia’ rather than as the main carer. Sentences such as ‘*Izan helps care for his grandma who is living with dementia…*’ or ‘*As a young carer you might or might not be involved in…*’
	A context of support: the programmed needs to present all possible and expected sources of support (across all modules)	▪ Support sources described: family members, friends, schools and teachers, young carers projects. ▪ Relevant web link with information on all Alzheimer Associations worldwide added at different stages of the programme.
**CONTENT** **(Illustrations)**	Patronizing (e.g., balloon images)	▪ Illustrations portraying human characters of young people and their family members added throughout (see Figure 3)
	Meaning hard to understand	▪ Meaningful symbols and other illustrations related to the content added (see Figure 4)
	Threatening (e.g., warning symbol in ‘Tip’ and ‘Remember’ text boxes)	▪ Warning symbols removed
	Cover image not appropriate for young people	▪ New cover image (see Figure 5)
**CONTENT** **(Narratives)**	Characters in case scenarios primarily older adults, often spouses	Case scenarios across five modules edited and new ones created: ▪ Placing the focus on a young character with caring responsibilities within a family unit. **Example:** ‘*Diana helps her mum take care of her dad Dan who has dementia. She is also trying to get good grades in school. Some days she feels really stressed with everything going on*’ (Module 3, Lesson 1). ▪ Describing situations that are familiar to young people. **Example:** ‘*Tom often feels tense when is on the school bus in the morning. The ride is about half an hour long and he starts worrying about all the schoolwork he has to do on top of helping his dad care for his mum. He decides to do a breathing exercise while on the bus’* (Module 3, Lesson 1). ▪ Presenting young people who support their family in caring for their family member rather than being the primary carer. **Example:** *‘Evie’s grandma, Martha, likes to go to the community centre every Saturday afternoon. Evie helps out by walking with Martha to the centre and then bringing her back home’* (Module 4, Lesson 5).
	Case scenarios describing activities or situations unlikely (or inappropriate) for a young person	
	No mention of schools and the role they might play	▪ New section ‘*How school can help*’ added in Module 2 (Lesson 1–The journey together) ▪ Case scenarios that discuss school topics added (e.g., going out with friends, homework) ▪ Illustrations of young people in school uniform added (see Figure 6).
	Lack of information regarding transition to care home (and the impact it might have on young carers)	▪ New section ‘*Planning for future care’* added in Module 2 (Lesson 1 – The journey together)
**LANGUAGE**	Too technical (complex)	**Example:** ‘*Transmitters*’ replaced by ‘*chemicals*’; ‘*Visual hallucinations*’ explained as ‘*seeing things that are not there*’; **‘***Delusions/Unreal thoughts’* replaced by ‘*strong belief not shared by others (unreal beliefs)*’; ‘*Distressing self-protected behaviours’* replaced by ‘*difficult behaviours’*
	Too formal or threatening	**Example:** ‘*Brain tumour’* deleted; ‘*How to plan pleasant activities*’ replaced by ‘*Making time for yourself*’; *‘Unmet needs’* replaced by *‘things you or the person living with dementia needs but you are struggling to achieve’; ‘Promote good sleep’* replaced by *‘help the person with dementia sleep better’; ‘Changes in judgement’* replaced by *‘saying the wrong thing at the wrong time’*
	Old fashioned	**Example:***‘Making a phone call’* replaced by *‘on your phone (mobile)’*
	Use of language that can sound ‘judgemental’ in the multiple-choice case scenario exercises	▪ Examples of replacements using tentative language: - ‘*This is the correct answer*’ replaced by ‘*This could be a helpful answer*’ - ‘*This answer is incorrect*’ replaced by ‘*This answer might not be very helpful*’ - ‘*Please select all answers that you think are correct*’ replaced by ‘*Please select all answers that you think might be appropriate/helpful/work best*’ ▪ Sentences with ‘Should’ replaced by ‘Would’ or ‘Could’ whenever possible
	Use of language that implies that everybody’s experience of living with dementia and their symptoms are the same	**Example:** ‘*People with dementia experience…*’ replaced by ‘*Sometimes people with dementia may experience…’* ‘Remember’ text box added: ‘*Just because a person has one or two of the symptoms listed in Activity 1 doesn’t mean they have dementia*’ (Module 1)
	In Module 3 (Lesson 3) there is only reference to pleasant activities	▪ This lesson has been edited to include the term meaningful. Meaningful activities (not always pleasurable) can also be important to young carers (e.g., doing homework)
	Language that sounds ‘too much like school’	▪ The word ‘*lesson*’ replaced to ‘*session*’ throughout ▪ ‘*Improve your skills…*’ replaced by ‘*we will talk about how you can learn about…’*

## Data Availability

If you wish to access the anonymized transcripts please email the corresponding author.

## Supplementary Materials

The following supporting information can be downloaded at: https://www.mdpi.com/article/10.3390/ijerph20010127/s1, Table S1: Structure and content of Workshop 1.

## References

[1] Burns A., Issacs J., Carter J. (2017). Tackling the Challenges of Young Onset Dementia. www.england.nhs.uk/blog/tackling-the-challenges-of-youngonset-dementia/.

[2] Hall M., Sikes P. (2020). ‘It’s just limboland’: Parental dementia and young people’s life courses. Sociol. Rev..

[3] Prince M., Wimo A., Guerchet M., Ali G.C., Wu Y.T., Prina M. (2015). World Alzheimer’s Report 2015, The Global Impact of Dementia: An Analysis of Prevalence, Incidence, Cost and Trends. https://www.alzint.org/u/WorldAlzheimerReport2015.pdf.

[4] World Health Organisation (2017). Global Action Plan on the Public Health Response to Dementia 2017–2025. https://www.who.int/publications/i/item/global-action-plan-on-the-public-health-response-to-dementia-2017-2025.

[5] Alzheimer’s Disease International (2020). From Plan to Impact III Maintaining Dementia as a Priority in Unprecedented Times. https://www.alzint.org/u/from-plan-to-impact-2020.pdf.

[6] Walter E., Pinquart M. (2020). How effective are dementia carer interventions? An updated comprehensive meta-analysis. Gerontol..

[7] Svanberg E., Stott J., Spector A. (2010). ‘Just Helping’: Children living with a parent with young onset dementia. Aging Ment. Health.

[8] Celdran M., Villar F., Triado C. (2012). When grandparents have dementia: Effects on their grandchildren’s family relationships. J. Fam. Issues.

[9] National Children’s Bureau 2016 Young People Caring for Adults with Dementia in England. https://www.basw.co.uk/system/files/resources/basw_105829-9_0.pdf.

[10] Santini S., Socci M., D’Amen B., Di Rosa M., Casu G., Hlebec V., Lewis F., Leu A., Hoefman R., Brolin R. (2020). Positive and Negative Impacts of Caring among Adolescents Caring for Grandparents. Results from an Online Survey in Six European Countries and Implications for Future Research, Policy and Practice. Int. J. Environ. Res. Public Health.

[11] Venters S., Jones C.J. (2021). The experiences of grandchildren who provide care for a grandparent with dementia: A systematic review. Dementia.

[12] Becker S. (2007). Global perspectives on children’s unpaid caregiving in the family: Research and policy on ‘Young Carers’ in the UK, Australia, the USA, and Sub-Saharan Africa. Glob. Soc. Policy.

[13] Joseph S., Sempik J., Leu A., Becker S. (2020). Young Carers Research, Practice and Policy: An Overview and Critical Perspective on Possible Future Directions. Adolesc. Res. Rev..

[14] Hutchinson K., Roberts C., Kurrle S., Daly M. (2016). The emotional well-being of young people having a parent with younger onset dementia. Dementia.

[15] Cartwright A.V., Stoner C.R., Pione R.D., Spector A. (2021). The experiences of those affected by parental young onset dementia: A qualitative systematic literature review. Dementia.

[16] Masterson-Algar P., Williams S. (2020). “Thrown Into the Deep End”: Mapping the Experiences of Young People Living in a Family Affected by a Neurological Condition. Qual. Health Res..

[17] Hall M., Sikes P. (2018). From “what the hell is going on?” to the “mushy middle ground” to “getting used to a new normal”: Young people’s biographical narratives around navigating parental dementia. Illn. Crisis Loss.

[18] Pakenham K.I., Cox S. (2018). Effects of benefit finding, social support and caregiving on youth adjustment in a parental illness context. J. Child Fam. Stud..

[19] HM Government Care Act 2014. https://www.legislation.gov.uk/ukpga/2014/23/contents/enacted.

[20] HM Government Health and Care Act 2022. https://www.legislation.gov.uk/ukpga/2022/31/contents/enacted.

[21] Svanberg E., Spector A., Stott J. (2011). The impact of young onset dementia on the family: A literature review. Int. Psychogeriatr..

[22] Kovaleva M., Blevins L., Griffiths P.C., Hepburn K. (2019). An Online Program for Caregivers of Persons Living With Dementia: Lessons Learned. J. Appl. Gerontol..

[23] Masterson-Algar P., Allen M.C., Hyde M., Keating N., Windle G. (2022). Exploring the impact of Covid-19 on the care and quality of life of people with dementia and their carers: A scoping review. Dementia.

[24] Naunton Morgan B., Windle G., Sharp R., Lamers C. (2022). eHealth and Web-Based Interventions for Informal Carers of People With Dementia in the Community: Umbrella Review. J. Med. Internet Res..

[25] O’Connell M.E., Crossley M., Cammer A., Morgan D., Allingham W., Cheavins B., Dalziel D., Lemire M., Mitchell S., Morgan E. (2014). Development and evaluation of a telehealth video-conferenced support group for rural spouses of individuals diagnosed with atypical early-onset dementias. Dementia.

[26] Rogers M.A.M., Lemmen K., Kramer R., Mann J., Chopra V. (2017). Internet-delivered health interventions that work: Systematic review of meta-analyses and evaluation of website availability. J. Med. Internet Res..

[27] Hassan A.Y.I. (2020). Challenges and recommendations for the deployment of information and communication technology solutions for informal caregivers: Scoping review. JMIR Aging.

[28] Sit H.F., Ling R., Lam A.I.F., Chen W., Latkin C.A., Hall B.J. (2020). The Cultural Adaptation of Step-by-Step: An Intervention to Address Depression Among Chinese Young Adults. Front. Psychiatry.

[29] Pot A.M., Gallagher-Thompson D., Xiao L.D., Willemse B.M., Rosier I., Mehta K.M., Zandi D., Dua T. (2019). iSupport: A WHO global online intervention for informal caregivers of people with dementia. World Psychiatry.

[30] Windle G., Flynn G., Hoare Z., Masterson-Algar P., Egan K., Edwards R.T., Jones C., Spector A., Algar-Skaife K., Hughes G. (2022). Effects of an e-health intervention ‘iSupport’ for reducing distress of dementia carers: Protocol for a randomised controlled trial and feasibility study. BMJ Open.

[31] Teles S., Napolskij M.S., Paúl C., Ferreira A., Seeher K. (2020). Training and support for caregivers of people with dementia: The process of culturally adapting the World Health Organization iSupport programme to Portugal. Dementia.

[32] Xiao L.D., McKechnie S., Jeffers L., Bellis A., Beattie E., Low L.F., Draper B., Messent P., Pot A.M. (2020). Stakeholders’ perspectives on adapting the World Health Organization iSupport for Dementia in Australia. Dementia.

[33] Baruah U., Loganathan S., Shivakumar P., Pot A.M., Mehta K.M., Gallagher-Thompson D., Dua T., Varghese M. (2021). Adaptation of an online training and support program for caregivers of people with dementia to Indian cultural setting. Asian J. Psychiatry.

[34] Power J., Gilmore B., Vallières F., Toomey E., Mannan H., McAuliffe E. (2019). Adapting health interventions for local fit when scaling-up: A realist review protocol. BMJ Open.

[35] Duggleby W., Peacock S., Ploeg J., Swindle J., Kaewwilai L., Lee H. (2020). Qualitative Research and Its Importance in Adapting Interventions. Qual. Health Res..

[36] World Health Organization (2017). iSupport Version 1.0. Adaptation and Implementation Guide.

[37] Teles S., Paúl C., Lima P., Chilro R., Ferreira A. (2021). User feedback and usability testing of an online training and support program for dementia carers. Internet Interv..

[38] Ottaviani A.C., Monteiro D.Q., Ferreira Campos C.R., Barham E.J., Oliveira D., da Cruz K.C.T., Corrêa L., de Souza Orlandi F., Zazzetta M.S., Gratão A.C.M. (2022). ISupport-Brasil: Preliminary results of the usability and acceptability assessment by caregivers of people who have dementia. Front. Med..

[39] Bernal G., Bonilla J., Bellido C. (1995). Ecological validity and cultural sensitivity for outcome research: Issues for the cultural adaptation and development of psychosocial treatments with Hispanics. J Abnorm. Child Psychol..

[40] HM Government Data Protection Act 2018. https://www.legislation.gov.uk/ukpga/2018/12/contents/enacted.

[41] Kuczynski L., Kuczynski L. (2003). Beyond bidirectionality. Bilateral conceptual frameworks for understanding dynamics in parent-child relations. Handbook of Dynamics in Parent-Child Relations.

[42] Cameron T.M., Walker M.F., Fisher R.J. (2022). A Qualitative Study Exploring the Lives and Caring Practices of Young Carers of Stroke Survivors. Int. J. Environ. Res. Public Health.

[43] Fives A., Kennan D., Canavan J., Brady B. (2013). Why we still need the term ‘young carer’: Findings from an exploratory study of young carers in Ireland. Crit. Soc. Work..

[44] Cassidy T., Giles M., McLaughlin M. (2014). Benefit finding and resilience in child caregivers. Br. J. Health Psychol..

[45] Stamatopoulos V. (2018). The young carer penalty: Exploring the costs of caregiving among a sample of Canadian youth. Child. Youth Serv..

[46] Hamilton M.G., Adamson E. (2013). Bounded agency in young carers’ life course-stage domains and transitions. J. Youth Stud..

[47] Phillips D., Paul G., Fahy M., Dowling-Hetherington L., Kroll T., Moloney B., Duffy C., Fealy G., Lafferty A. (2020). The invisible workforce during the COVID-19 pandemic: Family carers at the frontline. HRB Open Res..

[48] Holthe T., Halvorsrud L., Karterud D., Hoel K.A., Lund A. (2018). Usability and acceptability of technology for community-dwelling older adults with mild cognitive impairment and dementia: A systematic literature review. Clin. Interv. Aging.

[49] Leng M., Zhao Y., Xiao H., Li C., Wang Z. (2020). Internet-based supportive interventions for family caregivers of people with dementia: Systematic review and meta-analysis. J Med. Internet Res..

[50] Barrera M., Castro F.G., Strycker L.A., Toobert D.J. (2013). Cultural adaptations of behavioral health interventions: A progress report. J. Consult. Clin. Psychol..

[51] Skivington K., Matthews L., Simpson S.A., Craig P., Baird J., Blazeby J.M., Boyd K.A., Craig N., French D.P., McIntosh E. (2021). A new framework for developing and evaluating complex interventions: Update of Medical Research Council guidance. BMJ.

[52] Stephan A., Bieber A., Hopper L., Joyce R., Irving K., Zanetti O., Portolani E., Kerpershoek L., Verhey F., De Vugt M. (2018). Barriers and facilitators to the access to and use of formal dementia care: Findings of a focus group study with people with dementia, informal carers and health and social care professionals in eight European countries. BMC Geriatr..

[53] Parra-Vidales E., Soto-Pérez F., Perea-Bartolomé M.V., Franco-Martín M.A., Muñoz-Sánchez J.L. (2017). Online interventions for caregivers of people with dementia: A systematic review. Actas Esp. Psiquiatr..

[54] Waller A., Dilworth S., Mansfield E., Sanson-Fisher R. (2017). Computer and telephone delivered interventions to support caregivers of people with dementia: A systematic review of research output and quality. BMC Geriatr..

[55] Oliveira D., Da Mata F., Mateus E., Musyimi C., Farina N., Ferri C., Evans-Lacko S. (2021). Experiences of stigma and discrimination among people living with dementia and family carers in Brazil: Qualitative study. Ageing Soc..

