# Supplementary material for: iSupport for Young Carers: An Adaptation of an e-Health Intervention for Young Dementia Carers
Source: Int J Environ Res Public Health. 2022 Dec 22;20(1):127. doi: 10.3390/ijerph20010127 (PMC9819145; doi:10.3390/ijerph20010127)
Supplement: Supplementary file 1 [file ijerph-20-00127-s001.zip › ijerph-2074541-supplementary.pdf]

Table S1: Structure and content of Workshop 1.

Workshop 1: ADAPTING iSupport TO YOUNG

CARERS Date: 21st February 2022

Plan for the day (Questions and Prompts):

**Welcome**	• Brief background information (Why are we here? Ground rules)
**1. Who are we?**	• Icebreakers: Brief introduction exercise to give the chance for each person to say a little bit about themselves.<br>ACTIVITY: using Play Dough please make anything you want that you think might represent you (e.g., the things you like, your personality, the food you like etc.)
**2. Why are we here?**	• Did you enjoy looking through the information on iSupport? Why?<br>• What did you like best?<br>• What did you like the least?<br>• Did you understand the information in it? Did you relate to it?
**3. Thinking about support networks**	ACTIVITY – Creating a Mind Map of support networks. Using colour pens and whiteboard think about where you go for support (who do you talk to? What do you do? Where?) (prior to the activity participants will be shown examples) – Open discussion about support networks.
**BREAK**	
**4. Thinking about 'caring' – PART 1**	• What is the nicest thing about helping care?<br>• Can you think of times when you have felt happy/not very happy about your caring role? Can you think about a particular situation?<br>• Can you do the things you like doing? Can you think of some examples?
**5. Thinking about 'caring' – PART 2**	School and friends<br>• How did school find out about your situation? Does your school and teachers know?<br>• What is the most helpful thing someone in school has done for you?<br>• Who in the school would you go to? Why?<br>• Do friends understand what you are experiencing? Do they know? How do you explain it to them?
**6. Having a think about illustrations**	• What do you think about the illustrations in iSupport? Do you think they are useful? Why? Would you change them? Why? What type of illustration would you prefer?
**7. Final conclusions**	• What next? What am I going to do with all the feedback you have given me?
